# Sex differences evident in elevated anxiety symptoms in multiple sclerosis, inflammatory bowel disease, and rheumatoid arthritis

**DOI:** 10.3389/fpsyt.2023.1260420

**Published:** 2023-11-22

**Authors:** Jerlin Joyees, Ruth Ann Marrie, Charles N. Bernstein, James M. Bolton, John D. Fisk, Lesley A. Graff, Carol Hitchon, Scott B. Patten, Kaarina Kowalec, for the CIHR team in defining the burden and managing the effects of psychiatric comorbidity in chronic immunoinflammatory disease, Ruth Ann Marrie, Lesley Graff, John R. Walker, Carol A. Hitchon, Lisa M. Lix, James Bolton, Jitender Sareen, Alan Katz, James J. Marriott, Alexander Singer, Renée El-Gabalawy, Christine A. Peschken, Ryan Zarychanski, Charles N. Bernstein, John D. Fisk, Scott B. Patten, Lindsay I. Berrigan

**Affiliations:** ^1^College of Pharmacy, Rady Faculty of Health Sciences, University of Manitoba, Winnipeg, MB, Canada; ^2^Department of Internal Medicine, Max Rady College of Medicine, Rady Faculty of Health Sciences, University of Manitoba, Winnipeg, MB, Canada; ^3^Department of Community Health Sciences, Max Rady College of Medicine, Rady Faculty of Health Sciences, University of Manitoba, Winnipeg, MB, Canada; ^4^IBD Clinical and Research Centre, Rady Faculty of Health Sciences, University of Manitoba, Winnipeg, MB, Canada; ^5^Department of Psychiatry, Max Rady College of Medicine Rady Faculty of Health Sciences, University of Manitoba, Winnipeg, MB, Canada; ^6^Nova Scotia Health and Departments of Psychiatry, Psychology and Neuroscience, and Medicine, Dalhousie University, Halifax, NS, Canada; ^7^Department of Clinical Health Psychology, Max Rady College of Medicine, Rady Faculty of Health Sciences, University of Manitoba, Winnipeg, MB, Canada; ^8^Section of Rheumatology, Max Rady College of Medicine, Rady Faculty of Health Sciences, University of Manitoba, Winnipeg, MB, Canada; ^9^Department of Community Health Sciences, Cumming School of Medicine, University of Calgary, Calgary, AB, Canada; ^10^Department of Medical Epidemiology and Biostatistics, Karolinska Institutet, Solna, Sweden

**Keywords:** anxiety, sex, immune-mediated inflammatory disease, multiple sclerosis, rheumatoid arthritis, inflammatory bowel disease

## Abstract

**Introduction:**

Immune-mediated inflammatory diseases (IMID), such as multiple sclerosis (MS), inflammatory bowel disease (IBD) or rheumatoid arthritis (RA) have high rates of elevated anxiety symptoms. This can may worsen functioning and increase IMID disease burden. The rate of and factors associated with elevated anxiety symptoms may differ between males and females, which, in turn can affect diagnosis and disease management. We evaluated whether the frequency and factors associated with comorbid elevated anxiety symptoms in those with an IMID differed by sex.

**Methods:**

Participants with an IMID (MS, IBD or RA) completed two anxiety measures (HADS, GAD-7). We used logistic regression to investigate whether sex differences exist in the presence of comorbid elevated anxiety symptoms or in the endorsement of individual anxiety items in those with an IMID.

**Results:**

Of 656 participants, females with an IMID were more likely to have elevated anxiety symptoms compared to males (adjusted odds ratio [aOR] 2.05; 95%CI: 1.2, 3.6). Younger age, higher depressive symptoms and income were also associated with elevated anxiety symptoms in IMID. Lower income in males with an IMID, but not females, was associated with elevated anxiety symptoms (aOR: 4.8; 95%CI: 1.5, 15.6). No other factors demonstrated a sex difference. Males had nearly twice the odds of endorsing restlessness on the GAD-7 (OR = 1.8, 95%CI: 1.07, 3.15) compared to females.

**Discussion:**

We found evidence for sex differences in the factors associated with experiencing elevated anxiety symptoms in those with an IMID. These findings could be helpful to sensitize clinicians to monitor for comorbid anxiety symptoms in males with an IMID.

## 1 Introduction

Immune-mediated inflammatory diseases (IMID), such as multiple sclerosis (MS), inflammatory bowel disease (IBD) or rheumatoid arthritis (RA), are conditions caused by immune dysregulation (1). These IMIDs are characterized by many similarities such as acute exacerbations and progressive disability. Management of these diseases involves immuno-modulatory and immunosuppressive drug therapies, although each disease may require unique drugs. The treatment goal for each disease is remission as these diseases are incurable. These diseases also have high rates of comorbidities including psychiatric disorders (2, 3). Medical comorbidity is highly prevalent in those with a wide range on psychiatric disorders such as schizophrenia, bipolar disease, and depression (4–6). A systematic review found that those with major depressive disorder had higher rates of development and worsening of cardiovascular disorders, autoimmune disorders, metabolic disorders, and central nervous system disorders (5). Mental health and physical health are not independent of each other as inadequately controlled psychiatric disorders or medications for them can cause worsening physical comorbidity (5, 6). Likewise, adequate treatment of physical health is needed to have a positive impact on mental health (5).

Elevated anxiety symptoms are common among people with an IMID (7–10); those with an IMID are also more likely to develop an anxiety disorder compared to the general population (3). Anxiety symptoms may worsen functioning and increase IMID disease burden and are associated with work and cognitive impairment (11–15). Anxiety symptoms are often under-recognized and are considered secondary after depression. Elevated symptoms of anxiety adversely affect quality of life even in the absence of a clinical anxiety disorder diagnosis (16). In the IMID population, it is important to screen and identify those most at risk of elevated anxiety symptoms to promptly manage them as they could worsen IMID disease or increase risk of other psychiatric disorders (11–15). Since males and females may have different risk factors for anxiety symptoms, identifying prevalent risk factors in each sex can better target screening.

The odds of developing elevated anxiety symptoms differs by sex in the general population, with females more likely compared to males (17). Similarly, across studies examining MS, RA or IBD individually, females are up to 3-fold more likely to experience elevated anxiety symptoms or be diagnosed with an anxiety disorder (18–21). General population studies have found age, body mass index (BMI), income, education, and smoking to be factors associated with increased risk of anxiety symptoms (22–24), with income, education, and smoking more strongly associated with anxiety among females than males (25, 26). However, it is currently unknown in the IMID population, whether males and females have different risk factors for elevated anxiety symptoms.

Identifying sex differences in the frequency and factors associated with elevated anxiety symptoms is critical because of the underlying biological differences between males and females, which in turn can affect diagnosis and disease management. Therefore, we aimed to examine whether the frequency and factors associated with elevated anxiety symptoms differed between females and males with IMIDs. We used the Hospital Anxiety and Depression Scale (HADS) and the Generalized Anxiety Disorders-7 (GAD-7) scale to measure current anxiety symptoms. We hypothesized that females with MS, IBD or RA would have a higher rate of elevated anxiety symptoms compared to males and that BMI, income, education, and smoking would be more strongly associated with elevated anxiety symptoms in females compared with males (26, 27). We also aimed to determine whether sex is associated with the specific items included in the self-report anxiety scales in those with an IMID, given the lack of any IMID studies. Collectively, the results of this study would be valuable clinically as it may improve clinician recognition of anxiety presentations for males and females with an IMID.

## 2 Materials and methods

### 2.1 Study setting and population

This was a cross-sectional assessment that utilized data from a prospective cohort study which was evaluated the frequency and effects of psychiatric comorbidities in adults with an IMID in the central Canadian province of Manitoba (28). Participants were enrolled in the study if they had a confirmed diagnosis of MS, RA, or IBD. People with MS had a definite diagnosis of MS based on the prevailing criteria at the time of their diagnosis (28). Those with RA had a definite diagnosis based on the 2010 American College of Rheumatology/European League Against Rheumatism (ACR/EULAR) Rheumatoid Arthritis Classification Criteria (29). Last, those with IBD had a diagnosis of either Crohn’s disease or ulcerative colitis based on established endoscopy, radiology, and histology criteria (30, 31). Participants were not screened for the presence or absence of psychiatric disorders prior to the study inclusion. Participants were recruited from community and tertiary care centres in Winnipeg, Manitoba, using posters, social media outlets, and self-help groups (28). They were also recruited through targeted methods such as contacting patients with RA and IBD during clinic visits and contacting participants in the Winnipeg MS clinic registry by phone and mail (28). Participants were ≥ 18 years of age, provided informed consent, and were sufficiently proficient in English to complete questionnaires.

The University of Manitoba Health Research Ethics Board (HS17706/H2014:201) approved the study. All procedures were performed in accordance with relevant named guidelines and regulations.

### 2.2 Measures

All data for this study was obtained at the first visit of the larger prospective study.

#### 2.2.1 Participant characteristics

Self-reported questionnaires were used to collect the following background characteristics at the baseline study visit: gender, date of birth, annual household income (in Canadian dollars), highest level of education attained, and smoking history (28). For querying gender, participants were given two options (female/male) and < 1% of respondents did not have a gender that matched biological sex, so going forward we will refer to sex and not gender. Annual household income was categorized into <$50,000, ≥$50,000 and declined to answer. Highest level of education attained was categorized into ≤high school and >high school. Those who had smoked ≥100 cigarettes in their lifetime were defined as ever smokers (32).

Research assistants measured the participant’s height and weight to calculate BMI (kg/m^2^). BMI was categorized into underweight-normal (BMI < 25 kg/m^2^), overweight (BMI 25–30 kg/m^2^) or obese (BMI > 30 kg/m^2^).

#### 2.2.2 Pain and fatigue effects

Pain and fatigue are common symptoms of IMIDs. Pain interference was evaluated using the Modified Pain Effects Scale, which is a validated 6-item questionnaire with total scores ranging from 6 to 30, with higher scores relating to greater effects of pain on day-to-day functioning (33, 34). Fatigue was evaluated using the Fatigue Impact Scale for Daily Use (D-FIS). This is a validated 8-item scale that measures the experience and effect of daily fatigue. Total scores range from 0 to 32, with higher scores indicating greater impact of fatigue (35).

#### 2.2.3 IMID characteristics

Disease duration was calculated from the year of disease onset for each IMID. In MS, current disease course was categorized as relapsing–remitting, secondary-progressive or primary progressive (28). MS disease progression was evaluated using the Expanded Disability Status Scale (EDSS), which measures disability based on a standardized neurological exam, with higher scores indicating more disability (36).

IBD was characterized using the Montreal Classification into different phenotypes of Crohn’s disease (CD) or ulcerative colitis (UC) (30). Disease activity in CD and UC were characterized using the Harvey Bradshaw Disease Activity Index (37) and Powell-Tuck Index (38), respectively, through administration by research staff. These indices assess symptoms over the past week with a score ≥ 5 indicating active disease (28).

RA disease progression was characterized using the patient-reported modified Health Assessment Questionnaire (mHAQ) (39). The mHAQ assesses functional status by measuring the degree of difficulty in performing eight daily activities, with higher scores indicative of worse functionality (39). RA disease progression was also evaluated by determining if joint erosions were present (28).

#### 2.2.4 Psychiatric symptoms

Anxiety symptoms were evaluated using the Generalized Anxiety Disorder-7 (GAD-7) and Hospital Anxiety Depression Scale (HADS) measured at the baseline study visit. We employed both scales as they were designed for use in different populations, though each has been validated for use in the three immune diseases studied here (7–9). The GAD-7 scale consists of 7 items, the responses for which are summed; total scores range from 0 to 21 (40). These individual items evaluated feelings of nervousness, worrying, relaxation, restlessness, irritability, and feeling afraid. The optimal cut-off score for identifying clinically significant elevation of anxiety symptoms using the GAD-7 scale is ≥10 in the general population (40). As compared to a semi-structured psychiatric interview, the optimal cut-off score for the GAD-7 scale is 7 in the MS population, 8 in the IBD population, and 9 in the RA population, with a sensitivity of 71–91% and a specificity of 72–83% in the IMID groups (7–9). The HADS comprises two subscales, one for anxiety (HADS-A) and one for depression (HADS-D), each consisting of 7 items with a total score of 0–21 (41). For HADS-A, the individual items evaluated feelings of being tense, frightened, worrying, relaxation, restlessness, and panic. The optimal cut-off score for identifying clinically significant elevation of anxiety symptoms is ≥11 in the general population (41). As compared to a semi-structured psychiatric interview, the optimal HADS-A cut-off score is ≥9 in each of the IMID populations, with a sensitivity of 77–82% and a specificity of 59–76% in the IMID groups (7–9). We defined elevated anxiety symptoms as anyone meeting either GAD-7 ≥ 7 for MS, GAD-7 ≥ 9 for RA, GAD-7 ≥ 8 for IBD; or HADS-A ≥ 9 for any IMID (7–9). The presence of elevated anxiety symptoms was defined as meeting or exceeding the cut-off for either scale, to enhance sensitivity. We also did a sub-analysis using population cut-offs and each scale separately to define elevated anxiety symptoms. We included the HADS-D score to capture depressive symptoms, which occur commonly with anxiety symptoms.

### 2.3 Statistical analysis

We described the socio-demographic, IMID disease, and mental health characteristics of the participants with IMID, those with clinically meaningful elevated anxiety symptoms (yes/no defined using both HADS-A and GAD-7 scales in combination) and those with elevated anxiety symptoms stratified by sex (females with elevated anxiety symptoms/males with elevated anxiety symptoms). We described these groups using either the median (interquartile range: IQR), mean (standard deviation: SD), or frequency (%), as appropriate and compared them using a student’s t-test (continuous variables) or chi-square test (categorical variables).

We used logistic regression to investigate whether elevated anxiety symptoms in those with IMIDs were associated with various factors selected *a priori* given their association with anxiety in the literature (23, 24, 42–44) (sex, age, BMI, annual household income, education level, smoking status, IMID disease type, HADS-D score). To determine if the association of these covariates with elevated anxiety symptoms differed by sex, we added interaction terms between these covariates and sex. We also stratified the regression models findings by sex. We used our primary definition of elevated anxiety symptoms (anyone meeting either GAD-7 ≥ 7 for MS, GAD-7 ≥ 9 for RA, GAD-7 ≥ 8 for IBD; or HADS-A ≥ 9 for any IMID). We then performed three sensitivity analyses by varying the anxiety symptom measure used: (1) HADS-A (HADS-A ≥ 9 for all IMIDs), (2) GAD-7 (GAD-7 ≥ 7 for MS, ≥9 for RA, ≥8 for IBD), and (3) both HADS-A and GAD-7 but with general population thresholds (HADS-A ≥ 11 or GAD-7 ≥ 10). We report the overlap (or intersection) between the various definitions of elevated anxiety symptoms.

Last, we compared females and males who reported any anxiety symptoms (individual GAD-7 or HADS-A symptoms ≥1) using a chi-square test and then considered whether sex was associated with specific anxiety symptoms using logistic regression, after adjustment for total anxiety scale score (either GAD-7 or HADS-A) or total anxiety scale score and HADS-D score.

Statistical analysis was performed using SPSS (v28) and *R* for Statistical Computing (v4.2.2). The statistical significance level was set at *p* ≤ 0.05. Missing data were not imputed.

## 3 Results

### 3.1 Participant characteristics

There were 656 participants with an IMID, of whom 75.5% were female (Table 1). The average age of participants was 51.7 years. Of all the participants, 59% had smoked at some point in their life (ever-smokers). 59.5% of participants had an annual household income of ≥$50,000. The median disease duration was 18 years (interquartile range: 10.0–29.0) across all IMIDs (Table 2). A similar proportion had MS (38.9%) or IBD (37.7%) and fewer had RA (23.5%). One-third of those with an IMID met the criteria for elevated anxiety symptoms (n = 220, 33.5%, Table 1), with most being female (81.4%). Participants with MS had the highest frequency of elevated anxiety symptoms (41.4%), followed by IBD (35.5%); just under one quarter of those with RA reported elevated anxiety (23.2%, Table 2). There were many similarities between those with and without elevated anxiety symptoms. Females were the majority of those with (81.4%) and without (72.5%) elevated anxiety symptoms (Table 1). Both groups were similar in age and had similar BMI (Table 1). However, more individuals in those with elevated anxiety symptoms reported lower income versus those without elevated anxiety symptoms (38.2% vs. 28.7%, Table 1). Additionally, those with elevated anxiety symptoms reported greater impact of pain and fatigue and higher HADS-D scores compared to those without elevated anxiety symptoms (Table 1).

**Table 1:** Sociodemographic and general health characteristics of the total cohort, those with elevated anxiety symptoms and those with elevated anxiety symptoms as stratified by sex.

	[1] Total	[2] Elevated anxiety symptoms	[3] No Elevated anxiety symptoms	[4] Females, with elevated anxiety symptoms^a^	[5] Males, with elevated anxiety symptoms^a^	*p* [4] vs. [5]
*N*	656 (100)	220 (33.5)	436 (66.5)	179 (27.3)	41 (6.3)	**<0.001**
Sex: Female	495 (75.5)	179 (81.4)	316 (72.5)	–	–	–
Age, y^b^	51.7 (14.1)	49.4 (13.4)	52.8 (14.3)	49.3 (13.4)	49.9 (13.8)	0.40
Annual household income ($CAD)						0.24
<$50,000	209 (31.9)	84 (38.2)	125 (28.7)	64 (35.8)	20 (48.8)	
≥$50,000	390 (59.5)	116 (52.7)	274 (62.8)	97 (54.2)	19 (46.3)	
Declined	57 (8.7)	20 (9.1)	37 (8.5)	18 (10.1)	2 (4.9)	
Highest education						0.43
≤High School	212 (32.3)	86 (39.1)	126 (28.9)	69 (38.5)	17 (41.5)	
>High School	444 (67.7)	134 (60.9)	310 (71.1)	110 (61.5)	24 (58.5)	
BMI (kg/m^2^) ^c^	26.9 (23.5–31.5)	27.1 (23.8–32.3)	26.8 (23.4–31.3)	27.1 (23.6–32.3)	27.4 (25.9–31.7)	0.23
Underweight-normal (<25)	230 (35.1)	71 (32.3)	159 (36.5)	63 (35.2)	8 (19.5)	0.15
Overweight (25–30)	217 (33.1)	74 (33.6)	143 (32.8)	55 (30.7)	19 (46.3)	
Obese (>30)	201 (30.6)	72 (32.7)	129 (29.6)	59 (33.0)	13 (31.7)	
Missing	8 (1.2)	3 (1.4)	5 (1.1)	2 (1.1)	1 (2.4)	
Ever-Smoker	387 (59.0)	150 (68.2)	237 (54.4)	120 (67.0)	30 (73.2)	0.29
Modified Pain Effects Score^d^	12 (8.0–17.0)	17 (13.0–21.0)	10 (7.0–14.0)	17 (13.0–20.3)	17 (13.0–22.0)	0.19
Daily Fatigue Impact Scale^e^	9 (3.0–16.0)	16 (10.3–21.8)	2 (0–3.0)	16 (10.0–22.0)	15 (11.0–21.0)	0.29

Data presented as *N* (%) for categorical and median (IQR) or mean (SD) for continuous variables. ^a^There were 220 total cases of anxiety, defined by either GAD-7 ≥ 7 for MS, GAD-7 ≥ 9 for RA, GAD-7 ≥ 8 for IBD, or HADS-A ≥ 9 for any IMID. ^b^Mean (SD) calculated. ^c^N = 8 missing BMI data. ^d^*N* = 5 missing Pain Effects Score. ^e^*N* = 1 missing Daily Fatigue Impact Score. The final value of *p* column compares column 4 vs. 5 using the appropriate statistical test (student’s *t*-test = continuous variables; chi-square test = categorical variables), with bolding demonstrating those *p* ≤ 0.05.

**Table 2:** Index disease and anxiety characteristics of the total cohort, comparing those with elevated anxiety symptoms and those with elevated anxiety symptoms, stratified by sex.

	[1] Total	[2] Elevated anxiety symptoms	[3] No Elevated anxiety symptoms	[4] Females, with elevated anxiety symptoms^a^	[5] Males, with elevated anxiety symptoms^a^	*p* [4] vs. [5]
*N*	656	220	436	179	41	
IMID type						
Multiple sclerosis	255 (38.9)	91 (41.4)	164 (37.6)	77 (43.0)	14 (34.1)	**<0.001**
Rheumatoid arthritis	154 (23.5)	51 (23.2)	103 (23.6)	48 (26.8)	3 (7.3)	**<0.001**
Inflammatory bowel disease	247 (37.7)	78 (35.5)	169 (38.8)	54 (30.2)	24 (58.5)	**<0.001**
IMID disease duration, y	18.0 (10.0–29.0)	18.0 (11.0–29.0)	19.0 (10.0–29.0)	18.0 (11.0–29.0)	17.0 (8.0–27.5)	0.14
*Multiple sclerosis-related*						
Disease course^b^						0.40
Relapsing–remitting	183 (71.8)	73 (80.2)	110 (67.1)	62 (80.5)	11 (78.6)	
Secondary-progressive	49 (19.2)	12 (13.2)	37 (22.6)	NR	NR	
Primary progressive	23 (9.0)	NR	17 (10.4)	NR	NR	
Expanded Disability Status Scale	4.0 (3.0–6.0)	4.0 (3.0–5.5)	4.0 (3.0–6.0)	3.5 (2.5–5.3)	4.0 (3.0–5.6)	0.19
*Rheumatoid arthritis-related*						
Modified Health Assessment Questionnaire	4.0 (0–6.5)	6.0 (2.8–9.0)	3.0 (0–6.0)	6.0 (3.0–9.0)	2.0 (0)	0.27
*Inflammatory bowel disease-related*						
Disease type						0.051
Ulcerative colitis	94 (38.1)	27 (34.6)	67 (39.6)	15 (27.8)	12 (50.0)	
Crohn’s disease	153 (61.9)	51 (65.4)	102 (60.4)	39 (72.2)	12 (50.0)	
Powell-Tuck Activity Score	2.5 (1.0–5.0)	4.0 (2.0–9.0)	2.0 (1.0–4.0)	4.0 (1.0–8.0)	6.5 (2.3–10.0)	0.09
Harvey Bradshaw Disease Activity Index	4.0 (1.0–6.0)	6.0 (3.0–7.0)	3.0 (1.0–6.0)	6.0 (3.8–7.0)	4.0 (1.0–7.0)	0.15
*Mental health*						
GAD-7						
GAD-7 score	3 (1.0–7.0)	10 (7.0–13.0)	2.0 (0.0–3.0)	10.0 (7.0–13.0)	10.0 (7.0–15.0)	0.49
GAD-7 ≥ 10	112 (17.2)	112 (51.9)	0 (0)	91 (51.4)	21 (53.9)	0.46
HADS						
HADS-A score	6.0 (3.0–9.0)	10 (9.0–12.0)	4.0 (2.0–6.0)	10 (9.0–13.0)	10.0 (9.0–12.0)	0.25
HADS-A ≥ 9	184 (28.2)	184 (84.0)	0 (0)	149 (83.7)	35 (85.4)	0.50
HADS-A ≥ 11	102 (15.6)	102 (46.6)	0 (0)	82 (46.1)	20 (48.8)	0.44
HADS-D score	4.0 (2.0–7.0)	7.0 (5.0–10.0)	2.0 (1.0–4.0)	7.0 (5.0–9.0)	8.0 (6.0–11.0)	**<0.001**

Data presented as *N* (%) for categorical and median (IQR) or mean (SD) for continuous variables. NR: not reported due to small cell size. ^a^*N* = 220 total cases of anxiety, defined by either GAD-7 ≥ 7 for MS, GAD-7 ≥ 9 for RA, GAD-7 ≥ 8 for IBD, or HADS-A ≥ 9 for any IMID. ^b^No clinically isolated syndrome. Bolding demonstrating those *p* ≤ 0.05.

### 3.2 Sex differences amongst those with an IMID and elevated anxiety symptoms

When comparing females and males with IMIDs regarding anxiety symptoms, regression analyses identified that females were more likely to have elevated anxiety symptoms compared to males (odds ratio [OR] for female 1.66; 95%CI 1.1, 2.5; *p* = 0.013; Table 3), which remained following adjusting for other factors including age, depressive symptoms and IMID type (adjusted OR [aOR] 2.05; 95%CI 1.2, 3.6; *p* = 0.01). In this adjusted model, younger age, and HADS-D score were also associated with elevated anxiety symptoms (*p* < 0.05). In the regression models including a sex interaction term, there was a statistically significant interaction only between sex and lower income (ß_males_ = 1.75, *p* = 0.007). In the sex-stratified models, males who had a lower income had ~4-fold higher odds of elevated anxiety symptoms. In contrast, income was not associated with elevated anxiety among females (aOR 0.79; *p* > 0.5) and both sexes had similarly significantly increased odds of elevated anxiety in the presence of elevated HADS-D scores (Table 3). In the sensitivity analyses when using either the HADS-A or GAD-7 alone, or when using a general population cut-off to define those with elevated anxiety symptoms, we found results similar in direction but smaller in magnitude to that of the primary analyses (Supplementary Tables S1–S3), likely due to the reduced sample size (Supplementary Figure S1).

**Table 3:** Regression results investigating factors associated with elevated anxiety symptoms for individuals with IMID and stratified by sex.

	All				Sex-stratified, fully adjusted			
	Unadjusted (*N* = 648–656)		Adjusted (*N* = 644)		Female (*n* = 487)		Male (*n* = 157)	
Factor	OR (95% CI)	*p*	OR (95% CI)	*p*	OR (95% CI)	*p*	OR (95% CI)	*p*
*Sex: Female*	1.66 (1.11–247)	**0.013**	2.05 (1.17–3.57)	**0.01**	N/A			
*Age, y*	0.98 (0.97–0.99)	**0.003**	0.97 (0.95–0.99)	**<0.001**	0.96 (0.94–0.98)	**<0.001**	0.98 (0.94–1.03)	0.60
*Body mass index*								
Underweight-normal	Ref.		Ref.		Ref.		Ref.	
Overweight	1.16 (0.78–1.72)	0.47	1.13 (0.68–1.89)	0.61	0.97 (0.55–1.72)	0.48	3.44 (0.82–14.42)	0.09
Obese	1.25 (0.84–1.87)	0.28	0.83 (0.49–1.41)	0.50	0.67 (0.38–1.19)	0.46	3.67 (0.74–18.3)	0.11
*IMID type*								
RA	Ref.		Ref.		Ref.		Ref.	
MS	1.12 (0.74–1.71)	0.59	1.05 (0.60–1.85)	0.83	0.82 (0.44–1.50)	0.23	5.53 (0.78–38.9)	0.08
IBD	0.93 (0.61–1.43)	0.75	1.22 (0.65–2.28)	0.52	0.88 (0.43–1.77)	0.06	6.39 (1.01–40.7)	0.051
IMID disease duration, y	1.00 (0.99–1.01)	0.94	1.01 (0.99–1.03)	0.24	1.02 (0.99–1.04)	0.07	0.99 (0.94–1.04)	0.77
*Highest education*								
≤High school	1.58 (1.12–2.22)	**0.009**	1.28 (0.81–2.00)	0.29	1.37 (0.82–2.29)	0.22	0.89 (0.29–2.65)	0.84
>High school	Ref.		Ref.		Ref.		Ref.	
*Household income*								
Declined	1.28 (0.71–2.29)	0.41	0.90 (0.42–1.98)	0.76	0.83 (0.48–1.41)	0.49	0.89 (0.30–8.53)	0.83
<$50,000	1.59 (1.12–2.26)	**0.010**	1.08 (0.62–1.71)	0.80	0.79 (0.34–1.87)	0.60	4.82 (1.49–15.6)	**<0.001**
≥$50,000	Ref.		Ref.		Ref.		Ref.	
*Ever smoker*	1.80 (1.28–2.53)	**<0.001**	1.49 (0.95–2.32)	0.07	1.45 (0.89–2.38)	0.13	1.32 (0.41–4.26)	0.68
*HADS-D score*	1.51 (1.42–1.62)	**<0.001**	1.53 (1.42–1.65)	**<0.001**	1.56 (1.43–1.69)	**<0.001**	1.51 (1.29–1.76)	**<0.001**

The effect sizes are for the presence of comorbid elevated anxiety symptoms in IMID (absence of anxiety is the reference). In the unadjusted model, the included *N* are as follows: *N* = 648 (BMI), 652 (disease duration), 656 (all others). Multiple sclerosis (MS), rheumatoid arthritis (RA), inflammatory bowel disease (IBD). Bolded value of *p*: statistically significant at *p* ≤ 0.05.

### 3.3 Sex differences amongst individual anxiety scale items in those with an IMID

Last, we investigated whether individual anxiety scale items in the HADS-A or GAD-7 scales were associated with sex in those with an IMID (Table 4). The GAD-7 item regarding restlessness (question 5: “Being so restless that it’s hard to sit still”) was significantly associated with sex, with males almost twice as likely (aOR = 1.8; 95%CI 1.07, 3.15) to endorse this question as compared to females. When additionally adjusting for HADS-D score, we found results similar in direction and size. There were no other significant differences by sex for any of the other GAD-7 or HADS-A items, including the comparable HADS-A question for restlessness.

**Table 4:** Regression analyses investigating the differences in the endorsement of individual anxiety scale items in females and males with an IMID.

Outcome	Total, *N* (%)	[1] Female, *N* (%)	[2] Male, *N* (%)	*p* [1 vs. 2]	OR for males (95%CI), *p*	OR for males (95%CI), *p*
*GAD-7 scale*						
Q1: “Feeling nervous, anxious, or on edge”	370 (56.4)	295 (59.6)	75 (46.6)	**0.005**	0.83 (0.49–1.41), 0.4	0.81 (0.51–1.28), 0.3
Q2: “Not being able to stop or control worrying”	291 (44.4)	234 (47.3)	57 (35.4)	**0.010**	0.88 (0.49–1.57), 0.6	0.79 (0.47–1.32), 0.3
Q3: “Worrying too much about different things”	373 (57.0)	295 (59.8)	78 (48.4)	**0.013**	0.88 (0.52–1.48), 0.6	0.89 (0.56–1.39), 0.5
Q4: “Trouble relaxing”	327 (50.0)	260 (52.6)	67 (41.9)	**0.023**	0.98 (0.57–1.68), 0.9	0.86 (0.53–1.38), 0.5
Q5: “Being so restless that it’s hard to sit still”	187 (28.6)	141 (28.5)	46 (28.8)	1.00	1.84 (1.07–3.15),**0.027**	1.69 (1.02–2.78),**0.04**
Q6: “Becoming easily annoyed or irritable”	401 (61.2)	313 (63.2)	88 (55.0)	0.07	1.09 (0.67–1.76), 0.7	0.93 (0.59–1.45), 0.7
Q7: “Feeling afraid as if something awful might happen”	216 (32.9)	179 (36.2)	37 (23.0)	**0.002**	0.62 (0.35–1.10), 0.1	0.63 (0.37–1.06), 0.08
*HADS-A scale*						
Q1: “I feel tense or wound up”	524 (80.1)	413 (83.8)	111 (68.9)	**<0.001**	0.58 (0.33–1.03), 0.06	0.59 (0.33–1.05), 0.07
Q3: “I get a frightened feeling as if something awful is about to happen”	356 (54.4)	289 (58.5)	67 (41.6)	**<0.001**	0.69 (0.41–1.18), 0.1	0.72 (0.42–1.23), 0.2
Q5: “Worrying thoughts go through my mind”	487 (74.4)	384 (77.7)	103 (64.0)	**<0.001**	0.80 (0.46–1.37), 0.4	0.81 (0.47–1.41), 0.5
Q7: “I can sit at ease and feel relaxed”	456 (69.6)	356 (72.1)	100 (62.1)	**0.018**	1.14 (0.68–1.92), 0.6	1.09 (0.63–1.89), 0.7
Q9: “I get a sort frightened feeling like butterflies in the stomach”	375 (57.3)	297 (60.2)	78 (48.4)	**0.010**	0.95 (0.60–1.50), 0.8	0.99 (0.62–1.59), 0.9
Q11: “I feel restless as if I have to be on the move”	437 (66.8)	339 (68.8)	98 (60.9)	0.068	1.14 (0.72–1.80), 0.5	1.16 (0.73–1.83), 0.5
Q13: “I get sudden feelings of panic”	373 (56.9)	297 (60.1)	76 (47.2)	**0.005**	0.92 (0.56–1.51), 0.7	0.94 (0.57–1.55), 0.8

The effect sizes are for the endorsement of the specific scale item, with adjustment for sex and total GAD-7 or HADS-A anxiety scale score or sex, total GAD-7 or HADS-A anxiety scale score and HADS-D score. Bolded value of *p*: statistically significant at *p* ≤ 0.05.

## 4 Discussion

Elevated anxiety symptoms in those with an IMID may worsen functioning and increase IMID disease burden (11) and adversely affect quality of life even in the absence of a clinical disorder diagnosis (16); thus, identifying those at risk of elevated anxiety symptoms is imperative to prevent the associated sequelae. Whether males and females with an IMID have different risk factors for elevated anxiety symptoms is currently unknown. Our study examined whether the prevalence and factors (age, BMI, annual household income, education level, smoking status, IMID disease type, depressive symptoms) associated with elevated anxiety symptoms in IMID differed by sex, and whether there were differences in endorsement of individual anxiety items on two commonly used self-report screening measures. We found that females with an IMID had a significantly higher odds for elevated anxiety symptoms, compared to males. Income was the only factor associated with elevated anxiety symptoms in which males had a differential effect compared to females. In terms of individual anxiety symptoms, the only sex difference was related to restlessness for males, and only for the GAD-7 and not for a similar item of the HADS-A.

As reported in previous studies using the HADS-A (45, 46) or GAD-7 (47, 48) in persons with an IMID, we found females with an IMID had higher odds of experiencing elevated anxiety symptoms compared to males. Studies in the general population using the same anxiety screening measures that we employed have also found similarly increased odds of elevated anxiety in females (49, 50). In the general population or the overall IMID population, the association between elevated anxiety symptoms and female sex has been clear, with consideration for similar confounders as that used here, including depressive symptoms and age (47, 51).

We found that the association of income with elevated anxiety symptoms in those with an IMID differed by sex. More specifically, males who had a lower income had ~4 times greater odds of elevated anxiety symptoms, whereas for females, there was no significant interaction for this factor. A previous MS study found an association with lower income and higher anxiety symptoms (52). However, this study and others (47, 53) on elevated anxiety symptoms in IMID did not stratify results by sex nor include a sex interaction term, highlighting the contribution of our study. We compared our results to the non-IMID, general population studies, where low income is a known risk factor for elevated anxiety symptoms (50). One study reported that in older adults, lower personal resources (for, e.g., financial resources) was associated more strongly in men than women for elevated anxiety symptoms (54). Given the cross-sectional nature of our study, a causal link between low income and elevated anxiety symptoms in males but not females cannot be drawn; but it can identify those who may require more careful consideration when screening for anxiety symptoms.

There were some unexpected results for our study, in that we did not find sex differences in some risk factors for elevated anxiety symptoms which are known from studies in the general population, including BMI. However, this could relate to the disease in question (IMID) or in the measurement of the factors. For example, research assistants measured BMI in our study participants, whereas previous studies reporting a female-BMI interaction for anxiety used a self-report BMI measure, which can be under or overestimated by sex (55).

We also investigated whether sex was associated with the endorsement of specific anxiety symptoms from the GAD-7 or HADS-A scales. Our study found females were more likely to endorse most items. However, when adjusting for total score of the applicable anxiety scale, with or without adjusting for depressive symptom score scales, sex did not differentiate endorsement of the individual anxiety items except for restlessness with males being more likely to report this. This was only found for the GAD-7 measure and not with the HADS-A. This was a novel analysis for sex differences, examining anxiety symptoms at the more granular level of individual items on self-report symptom scales in persons with an IMID. Overall, there was very little meaningful differences between sexes regarding specific anxiety items. Considering these were both brief screening measures for clinical anxiety, it is possible that longer measures with more detail of anxiety presentation may have demonstrated sex differences in the IMID samples, as sex differences in clinical presentation of anxiety have been found in the general population. In the general population, females are more likely to exhibit symptoms of fatigue, fear, muscle tension, and physical complaints compared to males (56).

These findings have clinical and research relevance. Clinically, physicians can be confident that the anxiety symptom complex presents relatively similar in males and females with an IMID. We only identified income as a differential risk factor between females and males; indicating that those for whom clinicians may warrant greater attention or earlier intervention. Preventative or treatment strategies for lessening anxiety symptoms, such as cognitive behavioral therapy, have been effective in MS, IBD and RA (57–59). In terms of research relevance, our findings identified income as a factor associated differentially between males and females with elevated anxiety symptoms, yet additional investigations are warranted. This was cross-sectional study; no casual inference can be made with the results. This highlights the need for longitudinal studies to establish temporal relationships between IMID, anxiety symptoms, sex, and income. In addition, future research could investigate whether there are differences in the longer-term outcomes of those with an IMID experiencing elevated anxiety symptoms, including persistence of symptoms.

There were strengths and limitations to this study. We investigated three different IMIDs to improve generalization of the findings within this class of conditions, rather than focusing on one condition. However, existing literature investigated individual IMIDs or did not include sex-stratification, which makes it difficult to generalize findings and highlights the contribution of this current work. We investigated whether there were sex differences in response to specific anxiety items, which was novel. The GAD-7 and HADS-A are reliable and validated anxiety scales that are commonly used measures of current anxiety symptoms. Lifetime clinical diagnoses of anxiety disorders may have different risk factors associated with them, but elevated symptoms of anxiety adversely affect quality of life even absent a clinical disorder diagnosis (16). A limitation to this study was that the number of participants in the individual IMID groups was relatively small, making it difficult to draw conclusions regarding sex differences in each IMID despite the relatively large, combined cohort. Furthermore, there was a relatively small proportion of males in our study compared to females, largely owning to the female preponderance of these IMIDs. We examined a limited number of factors associated with elevated anxiety and we did not consider medication use in our analysis, and this represents a future potential direction. However, we considered factors that were commonly considered in general population studies such as BMI and income and considered IMID-specific factors such as disease duration. Literature comparisons to our study findings were additionally challenging given that many studies inadequately differentiate the terms sex and gender, however more recent studies are using these terms more appropriately (60).

In conclusion, income was differentially associated with elevated anxiety symptoms in males and females with an IMID. While it is known that females more commonly present with elevated anxiety, our study identified that males with lower income were at elevated risk for anxiety symptoms, which can be helpful to sensitize clinicians to monitor for comorbid anxiety symptoms for males as well. Future studies with larger numbers in each of the IMID groups examined here, as well as other IMIDs, and incorporating a longitudinal design to investigate the relationship overtime would be beneficial in drawing further conclusions. As well, including younger participants may make future studies more applicable to a wider population.

## Data availability statement

The datasets presented in this article are not readily available because the datasets generated and/or analyzed during the current study are not available due to some participants not consenting to their data being shared outside the original study. For those participants that did consent to their data being shared outside the original study, reasonable requests for access to their data can be made to RM with the appropriate ethical approvals and data sharing agreements. Requests to access the datasets should be directed to rmarrie@hsc.mb.ca.

## Ethics statement

The studies involving humans were approved by University of Manitoba Health Research Ethics Board (HS17706/H2014:201). The studies were conducted in accordance with the local legislation and institutional requirements. The participants provided their written informed consent to participate in this study.

## CIHR team in defining the burden and managing the effects of psychiatric Comorbidity in Chronic Immunoinflammatory Disease

Ruth Ann Marrie, MD, PhD; Lesley Graff, PhD; John R. Walker PhD, Carol A. Hitchon, MSc, MD, Lisa M. Lix, PhD, James Bolton, MD, Jitender Sareen, MD, Alan Katz, MSc, MBChB, James J. Marriott, MSc, MD, Alexander Singer, MBBChBAO, Renée El-Gabalawy, PhD, Christine A. Peschken, MD, Ryan Zarychanski, MSc, MD, Charles N. Bernstein, MD, University of Manitoba, Winnipeg, MB, Canada; John D. Fisk, PhD, Dalhousie University, Winnipeg, MB, Canada; Scott B. Patten, MD, PhD, University of Calgary, Calgary, AB, Canada; Lindsay I. Berrigan, PhD, St. Francis Xavier University, Antigonish, NS, Canada.

## Author contributions

JJ: Formal analysis, Writing – original draft. RM: Conceptualization, Data curation, Funding acquisition, Methodology, Resources, Writing – review & editing. CB: Resources, Writing – review & editing. JB: Funding acquisition, Writing – review & editing. JF: Funding acquisition, Resources, Writing – review & editing. LG: Funding acquisition, Resources, Writing – review & editing. CH: Funding acquisition, Resources, Writing – review & editing. SP: Funding acquisition, Writing – review & editing. KK: Conceptualization, Formal analysis, Methodology, Software, Supervision, Visualization, Writing – original draft, Writing – review & editing.
