# Supplementary material for: Sex differences evident in elevated anxiety symptoms in multiple sclerosis, inflammatory bowel disease, and rheumatoid arthritis
Source: Front Psychiatry. 2023 Nov 22;14:1260420. doi: 10.3389/fpsyt.2023.1260420 (PMC10702748; doi:10.3389/fpsyt.2023.1260420)
Supplement: Supplementary file 1 [file Table_1.docx]

**Supplementary Information**

***Table S1:*** Sensitivity regression analysis with elevated anxiety symptoms being defined only by a HADS-A≥ 9.

|  | **All** | | | | **Sex-stratified, fully adjusted** | | | |
| --- | --- | --- | --- | --- | --- | --- | --- | --- |
|  | **Unadjusted (N=645-653)** | | **Adjusted (N=641)** | | **Female (n=484)** | | **Male (n=157)** | |
| **Factor** | **OR**  **(95% CI)** | **P** | **OR**  **(95% CI)** | **P** | **OR**  **(95% CI)** | **P** | **OR**  **(95% CI)** | **P** |
| *Sex: Female* | 1.56  (1.03-2.38) | **.037** | 1.86  (1.08-3.21) | **.025** | N/A | | | |
| *Age, y* | 0.99  (0.97-1.00) | **.013** | 0.97  (0.95-0.99) | **.03** | 0.97  (0.95-0.99) | **<.001** | 0.99  (0.95-1.04) | .88 |
| *Body mass index* | |  |  |  |  |  |  |  |
| Underweight-normal | Ref. |  | Ref. |  | Ref. |  | Ref. |  |
| Overweight | 1.28  (0.85-1.94) | .24 | 1.26  (0.76-2.08) | .35 | 1.03  (0.59-1.82) | .91 | 3.36  (0.89-12.8) | .07 |
| Obese | 1.13  (0.74-1.73) | .58 | 0.76  (0.45-1.29) | .32 | 0.71  (0.41-1.27) | .52 | 1.19  (0.23-6.08) | .21 |
| *IMID type* |  |  |  |  |  |  |  |  |
| RA | Ref. |  | Ref. |  | Ref. |  | Ref. |  |
| MS | 0.89  (0.58-1.38) | .61 | 0.78  (0.45-1.35) | .37 | 0.63  (0.35-1.15) | .48 | 2.68  (0.45-15.8) | .30 |
| IBD | 0.81  (0.52-1.26) | .35 | 0.94  (0.51-1.73) | .85 | 0.69  (0.35-1.37) | .30 | 3.55  (0.69-18.5) | .13 |
| IMID disease duration, y | 1.0  (0.99-1.01) | .85 | 1.00  (0.98-1.03) | .47 | 1.01  (0.99-1.04) | .15 | 0.98  (0.93-1.03) | .49 |
| *Highest education* | |  |  |  |  |  |  |  |
| ≤High school | 1.58  (1.12-2.25) | **.012** | 1.33  (0.85-2.10) | .20 | 1.54  (0.93-2.54) | .09 | 0.71  (0.24-2.11) | .53 |
| >High school | Ref. |  | Ref. |  | Ref. |  | Ref. |  |
| *Household income* | |  |  |  |  |  |  |  |
| Declined | 1.03  (0.55-1.94) | .93 | 0.64  (0.28-1.43) | .27 | 0.61  (0.26-1.43) | .31 | 0.35  (0.03-4.42) | .42 |
| <$50 000 | 1.43  (0.99-2.07) | .057 | 0.93  (0.58-1.48) | .76 | 0.76  (0.45-1.29) | .95 | 2.42  (0.80-7.34) | .11 |
| >$50 000 | Ref. |  | Ref. |  | Ref. |  | Ref. |  |
| *Ever smoker* | 1.65  (1.15-2.36) | **.006** | 1.38  (0.89-2.15) | .14 | 1.26  (0.78-2.05) | .35 | 1.85  (0.59-5.73) | .28 |
| *HADS-D score* | 1.41  (1.32-1.49) | **<.001** | 1.42  (1.32-1.51) | **<.001** | 1.42  (1.32-1.54) | **<.001** | 1.40  (1.22-1.61) | **<.001** |

^a^The effect sizes are for the presence of elevated anxiety symptoms in IMID using the HADS-A scale with a cut-off score of ≥9. In the unadjusted model, the included N are as follows: N=645 (BMI), 649 (disease duration), 653 (all others). We added an interaction term for sex and each factor listed to the adjusted model for the analyses, age ß=0.02, P=0.26, BMI-Overweight ß=1.17, P=0.11; BMI-Obese ß=0.50, P=0.56; IMID-MS ß=1.45, P=0.12; IMID-IBD ß=1.63, P=0.07; IMID disease duration ß=-0.03, P=0.23; Education-less than High School ß=-0.78,P=0.21; Income-<$50,000 ß=1.16, P=0.06; Income-Decline ß=-0.5, P=0.68; Smoking ß=0.38, P=0.54; HADS-D ß=-0.17, P=0.83. Bolded p-value: statistically significant at P≤0.05.
