# Supplementary material for: Sex differences evident in elevated anxiety symptoms in multiple sclerosis, inflammatory bowel disease, and rheumatoid arthritis
Source: Front Psychiatry. 2023 Nov 22;14:1260420. doi: 10.3389/fpsyt.2023.1260420 (PMC10702748; doi:10.3389/fpsyt.2023.1260420)
Supplement: Supplementary file 2 [file Table_2.docx]

**Supplementary Information**

***Table S2*:** Sensitivity regression analysis with elevated anxiety symptoms being defined by a GAD-7 ≥7 for multiple sclerosis, ≥9 for rheumatoid arthritis, or ≥8 for inflammatory bowel disease.

|  | **All** | | | | **Sex-stratified, fully adjusted** | | | |
| --- | --- | --- | --- | --- | --- | --- | --- | --- |
|  | **Unadjusted (N=642-650)** | | **Adjusted (N=638)** | | **Female (n=484)** | | **Male (n=154)** | |
| **Factor** | **OR**  **(95% CI)** | **P** | **OR**  **(95% CI)** | **P** | **OR**  **(95% CI)** | **P** | **OR**  **(95% CI)** | **P** |
| *Sex: Female* | 1.65  (1.06-2.57) | **.028** | 2.26  (1.21-4.22) | **<.001** | N/A | | | |
| *Age, y* | 0.98  (0.97-0.99) | **.001** | 0.97  (0.95-0.98) | **.01** | 0.97  (0.95-0.99) | **.001** | 0.96  (0.92-1.01) | .20 |
| *Body mass index* | |  |  |  |  |  |  |  |
| Underweight-normal | Ref. |  | Ref. |  | Ref. |  | Ref. |  |
| Overweight | 1.12  (0.72-1.71) | .65 | 1.02  (0.58-1.78) | .94 | 1.06  (0.57-1.99) | .94 | 1.26  (0.28-5.62) | .13 |
| Obese | 1.35  (0.88-2.09) | .17 | 0.86  (0.49-1.52) | .62 | 0.70  (0.37-1.31) | .37 | 3.15  (0.56-17.6) | .28 |
| *IMID type* |  |  |  |  |  |  |  |  |
| RA | Ref. |  | Ref. |  | Ref. |  | Ref. |  |
| MS | 1.56  (0.98-2.48) | .06 | 1.81  (0.97-3.37) | .06 | 1.56  (0.79-3.06) | .75 | 3.17  (0.41-24.4) | .31 |
| IBD | 1.02  (0.63-1.66) | .94 | 1.49  (0.74-2.98) | .25 | 1.41  (0.65-3.04) | .39 | 1.78  (0.26-11.9) | .23 |
| IMID disease duration, y | 0.99  (0.98-1.01) | .28 | 1.00  (0.97-1.02) | .98 | 1.00  (0.98-1.03) | .75 | 0.99  (0.94-1.05) | .82 |
| *Highest education* | |  |  |  |  |  |  |  |
| ≤High school | 1.59  (1.11-2.30) | **.013** | 1.22  (0.74-2.00) | .43 | 1.16  (0.66-2.04) | .23 | 1.25  (0.41-3.91) | .44 |
| >High school | Ref. |  | Ref. |  | Ref. |  | Ref. |  |
| *Household income* | |  |  |  |  |  |  |  |
| Declined | 1.46  (0.79-2.71) | .23 | 0.97  (0.57-1.61) | .89 | 0.84  (0.47-1.51) | .99 | 1.25  (0.09-16.8) | .86 |
| <$50 000 | 1.41  (0.97-2.07) | .08 | 1.05  (0.44-2.44) | .92 | 0.97  (0.38-2.44) | .65 | 2.26  (0.66-7.66) | .19 |
| ≥$50 000 | Ref. |  | Ref. |  | Ref. |  | Ref. |  |
| *Ever smoker* | 2.14  (1.46-3.13) | **<.001** | 1.96  (1.19-3.23) | **<.001** | 1.89  (1.09-3.28) | **<.001** | 2.33  (0.63-8.52) | .90 |
| *HADS-D score* | 1.51  (1.42-1.62) | **<.001** | 1.53  (1.42-1.66) | **<.001** | 1.58  (1.44-1.73) | **<.001** | 1.44  (1.24-1.69) | **<.001** |

^a^The effect sizes are for the presence of comorbid anxiety in IMID using the GAD-7 cut-off score of ≥7 (MS), ≥9 (RA), and ≥8 (IBD). In the unadjusted model, the included N are: N=642 (BMI), 646 (disease duration), 650 (all others). We added an interaction term for sex and each factor listed to the adjusted model for the analyses: age ß=-0.007, P=0.24; BMI-Overweight ß=0.16, P=0.83; BMI-Obese ß=1.50, P=0.11; IMID-MS ß=0.71, P=0.5; IMID-IBD ß=0.24, P=0.8; IMID disease duration ß=-0.01, P=0.7; Education-≤High School ß=0.07, P=0.9; Income-<$50,000 ß=0.97, P=0.15; Income-Declined ß=0.26, P=0.8; Smoking ß=0.21, P=0.7, HADS-D ß=0.09, P=0.3. Bolded p-value: statistically significant at P≤0.05.
