# Supplementary material for: Sex differences evident in elevated anxiety symptoms in multiple sclerosis, inflammatory bowel disease, and rheumatoid arthritis
Source: Front Psychiatry. 2023 Nov 22;14:1260420. doi: 10.3389/fpsyt.2023.1260420 (PMC10702748; doi:10.3389/fpsyt.2023.1260420)
Supplement: Supplementary file 3 [file Table_3.docx]

**Supplementary Information**

***Table S3:*** Sensitivity regression analysis with elevated anxiety symptoms being defined by either HADS-A ≥11 or GAD-7 ≥10 (population cut-offs).

|  | **All** | | | | **Sex-stratified, fully adjusted** | | | |
| --- | --- | --- | --- | --- | --- | --- | --- | --- |
|  | **Unadjusted (N=648-656)** | | **Adjusted (N=644)** | | **Female (n=487)** | | **Male (n=157)** | |
| **Factor** | **OR**  **(95% CI)** | **P** | **OR**  **(95% CI)** | **P** | **OR**  **(95% CI)** | **P** | **OR**  **(95% CI)** | **P** |
| *Sex: Female* | 1.47  (0.93-2.32) | .10 | 1.87  (1.02-3.43) | **.04** | N/A | | | |
| *Age, y* | 0.98  (0.97-0.996) | **.009** | 0.97  (0.95-0.98) | **.01** | 0.97  (0.95-0.99) | **.01** | 0.97  (0.93-1.02) | .27 |
| *Body mass index* | |  |  |  |  |  |  |  |
| Underweight-normal | Ref. |  | Ref. |  | Ref. |  | Ref. |  |
| Overweight | 1.48  (0.94-2.34) | .09 | 1.45  (0.83-2.53) | .07 | 1.44  (0.76-2.71) | .20 | 1.92  (0.47-7.82) | .53 |
| Obese | 1.33  (0.83-2.12) | .24 | 0.86  (0.48-1.54) | .31 | 0.74  (0.39-1.42) | .30 | 1.64  (0.32-8.46) | .13 |
| *IMID type* |  |  |  |  |  |  |  |  |
| RA | Ref. |  | Ref. |  | Ref. |  | Ref. |  |
| MS | 1.09  (0.67-1.75) | .74 | 0.99  (0.54-1.83) | .24 | 0.84  (0.43-1.65) | .64 | 2.28  (0.34-15.8) | .35 |
| IBD | 0.92  (0.56-1.50) | .73 | 1.09  (0.56-2.12) | .11 | 0.92  (0.42-1.97) | .83 | 2.42  (0.41-14.2) | .38 |
| IMID disease duration, y | 1.00  (0.98-1.01) | .56 | 1.00  (0.98-1.03) | .42 | 1.01  (0.98-1.04) | .24 | 1.00  (0.95-1.05) | .69 |
| *Highest education* | |  |  |  |  |  |  |  |
| ≤High school | 1.49  (1.01-2.18) | **.042** | 1.23  (0.74-2.00) | .11 | 1.37  (0.78-2.41) | .23 | 0.88  (0.29-2.65) | .80 |
| >High school | Ref. |  | Ref. |  | Ref. |  | Ref. |  |
| *Household income* | |  |  |  |  |  |  |  |
| Declined | 1.13  (0.58-2.19) | .72 | 0.74  (0.47-1.31) | .40 | 0.73  (0.42-1.31) | .21 | 1.15  (0.35-3.71) | .95 |
| <$50 000 | 1.23  (0.83-1.84) | .31 | 0.72  (0.30-1.70) | .96 | 0.57  (0.21-1.51) | .32 | 1.25  (0.15-10.0) | .15 |
| ≥$50 000 | Ref. |  | Ref. |  | Ref. |  | Ref. |  |
| *Ever smoker* | 1.81  (1.22-2.68) | **.003** | 1.54  (0.94-2.53) | .22 | 1.32  (0.76-2.28) | .34 | 2.82  (0.83-9.8) | .12 |
| *HADS-D score* | 1.45  (1.35-1.55) | **<.001** | 1.46  (1.37-1.56) | **<.001** | 1.52  (1.39-1.66) | **<.001** | 1.34  (1.15-1.53) | **<.001** |

^a^The effect sizes are for the presence of comorbid anxiety in IMID using population cut-offs for GADS-7 and HADS-A scales. In the unadjusted model, the included N are as follows: N=648 (BMI), 652 (disease duration), 656 (all others). We added an interaction term for sex and each factor listed to the adjusted model for the analyses, age ß=0.007,P=0.78; BMI-Overweight ß=0.29,P=0.4; BMI-Obese ß=0.79, P=0.4; IMID-MS ß=0.99, P=0.5; IMID-IBD ß=0.97, P=0.6; IMID disease duration ß=-0.01, P=0.7; Education-≤High School ß=-0.43, P=0.5; Income-<$50,000 ß=0.78, P=0.3; Income-Declined ß=0.46, P=0.4; Smoking ß=0.76, P=0.5, HADS-D ß=-0.13, P=0.1. Bolded p-value: statistically significant at P≤0.05.
